# Impact of *IL28B*-Related Single Nucleotide Polymorphisms on Liver Histopathology in Chronic Hepatitis C Genotype 2 and 3

**DOI:** 10.1371/journal.pone.0029370

**Published:** 2012-01-13

**Authors:** Karolina Rembeck, Åsa Alsiö, Peer Brehm Christensen, Martti Färkkilä, Nina Langeland, Mads Rauning Buhl, Court Pedersen, Kristine Mørch, Johan Westin, Magnus Lindh, Kristoffer Hellstrand, Gunnar Norkrans, Martin Lagging

**Affiliations:** 1 Department of Infectious Diseases/Virology, Institute of Biomedicine, University of Gothenburg, Gothenburg, Sweden; 2 Department of Infectious Diseases, University of Southern Denmark, Odense, Denmark; 3 Department of Gastroenterology, Helsinki University, Helsinki, Finland; 4 Department of Medicine, Haukeland University Hospital, Bergen, Norway; 5 Institute of Medicine, University of Bergen, Bergen, Norway; 6 Department of Infectious Diseases, Aarhus University, Aarhus, Denmark; University of Montreal, Canada

## Abstract

**Background and Aims:**

Recently, several genome-wide association studies have revealed that single nucleotide polymorphisms (SNPs) in proximity to *IL28B* predict spontaneous clearance of HCV infection as well as outcome following peginterferon and ribavirin therapy among HCV genotype 1 infected patients. The present study aimed to evaluate the impact of *IL28B* SNP variability on liver histology in the context of a phase III treatment trial (NORDynamIC) for treatment-naïve patients with chronic HCV genotype 2 or 3 infection, where pretreatment liver biopsies were mandatory.

**Methods:**

Three hundred and thirty-nine Caucasian patients had samples available for *IL28B* genotyping (*rs12979860*) of whom 314 had pretreatment liver biopsies that were evaluated using the Ishak protocol, allowing for detailed grading and staging of liver histopathology.

**Results:**

*IL28B* CC*_rs12979860_* genotype in HCV genotype 3 infected patients was associated with higher ALT levels (p<0.0001), higher AST to platelet ratio index (APRI; p = 0.001), and higher baseline viral load (p<0.0001) as compared to patients with the CT or TT genotypes. Additionally the CC*_rs12979860_* genotype entailed more pronounced portal inflammation (p = 0.02) and steatosis (p = 0.03). None of these associations were noted among HCV genotype 2 infected patients.

**Conclusion:**

This study shows that the CC*_rs12979860_* SNP is associated with more pronounced liver histopathology in patients chronically infected with HCV genotype 3, which may be secondary to higher viral load. The finding that *IL28B* variability did not impact on liver pathology or viral load among genotype 2 infected patients implies that *IL28B* may differentially regulate the course of genotype 2 and 3 infection.

## Introduction

Hepatitis C virus (HCV) infects 170 million people worldwide [1] and is a leading cause of chronic hepatitis, cirrhosis, and hepatocellular carcinoma [2]. Treatment with pegylated interferon-α (peg-IFN) and ribavirin results in sustained viral response (SVR) in approximately 50% of patients infected with HCV of genotype 1 and in 80% of those with HCV genotypes 2 or 3 [3], [4], [5]. Recently, several genome-wide association studies have revealed that single nucleotide polymorphisms (SNPs) in the *19q13* region, in close proximity to three genes (*IL28A*, *IL28B*, and *IL29*) encoding cytokines of the IFN-λ (i.e. type III IFN) family, predict spontaneous clearance of HCV infection [6], [7] as well as SVR following peg-IFN/ribavirin therapy among patients infected with HCV genotype 1 [6], [8], [9], [10]. Additionally, these polymorphisms are strongly associated with the first phase viral decline (i.e. reduction of HCV RNA during the first days of treatment) irrespective of HCV genotype [11], and concomitant assessment of pretreatment levels of systemic IP-10 and *IL28B*-related SNPs augments the prediction of the first phase decline in HCV RNA and the final therapeutic outcome [12], [13].

Carriage of the favorable C allele at *rs12979860* also is associated with higher baseline viral load (8, 11, 14), which otherwise is an established negative predictor of response to peg-IFN/ribavirin therapy [3], [4], [5], possibly secondary to major allele carriage being associated with lower intrahepatic expression of interferon-stimulated genes (ISGs) [14], [15]. A similarly unexpected observation is that a C allele at *rs12979860* is more common in Caucasians infected with HCV genotype 2 and 3 than with genotype 1 [13], [16], [17].

In a study of Japanese patients infected with HCV genotype 1 or 2, those homozygous for the *IL28B* major allele had significantly higher inflammatory activity; also, a higher proportion of these patients had fibrosis stage F2-4 as compared with F0-1, but interestingly these patients also had significantly lower gamma-GTP levels [18]. Similarly, among HCV genotype 3 infected patients of Scandinavian descent, CC carriers at *rs12979860* had significantly higher normalized alanine aminotransferase (ALT) levels as well as aspartate aminotransferase platelet ratio index (APRI) than T allele carriers indicating a higher degree of inflammation and fibrosis [19]. The aim of the present study was to evaluate the impact of *IL28B* SNP variability on liver histology in the context of a phase III treatment trial for treatment naïve patients with chronic HCV genotype 2 or 3 infection. In this trial, pretreatment liver biopsies were mandatory and were centrally evaluated using the Ishak protocol, allowing for detailed grading and staging of liver histopathology.

## Materials and Methods

### The NORDynamIC trial

Three hundred and eighty-two treatment naïve patients with HCV genotype 2/3 infection were randomized at baseline to either 12 or 24 weeks of combination treatment with peg-IFN α-2a 180 µg once weekly and ribavirin 800 mg daily. Baseline characteristics of the 339 Caucasian patients that could be analyzed for *IL28B* polymorphisms are detailed in Table 1. The first dose of study medication was administered under the supervision of a study nurse, and a patient diary monitored subsequent dosing. Further details regarding this trial are provided elsewhere [20].

**Table 1:** Baseline Characteristics with Patients Grouped According to HCV genotype and *IL28B* Genetic Variations (*rs12979860*).

	HCV genotype 2				HCV genotype 3			
	CC	CT	TT	P	CC	CT	TT	P
	n = 48	n = 40	n = 10		n = 101	n = 113	n = 27	
Age (years)^a^	49 (41–54)	47 (41–56)	48 (35–53)	NS	41 (32–48)	40 (33–48)	40 (29–46)	NS
Gender (Male/Female)^b^	28/20	24/17	6/4	NS	69/37	68/49	14/13	NS
BMI (kg/m^2^)^a^	25 (22–27)	25 (24–28)	25 (23–26)	NS	25 (23–28)	26 (23–29)	27 (23–28)	NS
Fibrosis Stage (Ishak 0/1/2/3/4/5/6)^b^	2/8/11/12/7/4/3	0/5/10/10/7/2/4	2/0/4/2/1/0/1	NS	2/8/33/28/9/7/10	3/17/36/26/16/4/6	1/6/6/5/3/2/0	NS
HOMA-IR^a^	2.7 (1.4–4.4)	2.4 (1.1–7.0)	2.5 (1.3–9.8)	NS	2.2 (1.4–5.0)	2.9 (1.9–6.6)	3.8 (2.0–7.7)	NS
Alkohol consumption (drinks/day)^a^	0.0 (0.0–0.5)	0.1 (0.0–0.4)	0.1 (0.0–0.4)	NS	0.1 (0.0–0.4)	0.0 (0.0–0.4)	0.0 (0.0–0.3)	NS

Data presented as median (25^th^–75th percentile)^a^ or n^b^.

### Classification of response

Patients were classified as having a rapid virological response (RVR) if HCV RNA was undetectable (i.e. <15 IU/mL) day 29, and as achieving SVR if plasma HCV RNA was undetectable 24 weeks after completion of therapy.

### *IL28B* genotyping

SNP *rs12979860* was determined in plasma by allelic discrimination using Taqman MGB (minor groove binding) probes. The following primers and probes were used: *rs12979860*: Forward, GTGCCTGTCGTGTACTGAACCA, Reverse, AGCGCGGAGTGCAATTCA, Probe_C, FAM-CCTGGTTCGCGCCTT-MGB, Probe_T, VICCCTGGTTCACGCCT-MGB. All SNPs were at Hardy-Weinberg equilibrium. SNP *rs12979860* has previously been reported to have a stronger association with both first phase decline and SVR than *rs8099917* and *rs12980275* among Caucasian HCV infected patients, and was thus analyzed in the present study [11].

### HCV RNA quantification

Plasma was obtained using PPT-tubes and HCV RNA was determined by RT-PCR of plasma using Cobas AmpliPrep/COBAS TaqMan HCV Test (Roche Diagnostics, Branchburg, NJ), which quantifies HCV RNA with a limit of detection of ≤15 IU/mL. HCV RNA quantification was performed on days 0, 3, 7, 8, 29, week 8, week 12, week 24 (for those receiving 24 weeks of therapy), and 24 weeks after completion of therapy. All samples were frozen (−70°C) and subsequently analyzed at the central laboratory.

### Liver biopsies

Liver biopsies were obtained from all patients within 24 months prior to study entry. Only biopsies with a length exceeding 1.5 cm and containing more than 6 portal tracts were evaluated. The evaluation was performed in a blinded fashion by two independent observers according to the Ishak protocol [21]. Equivocal issues were debated after the independent scores were noted, and a consensus score was obtained. In addition, steatosis was graded as follows: absent = 0, less than 30% of hepatocytes involved = 1, 30–70% of hepatocytes involved = 2, and more than 70% of hepatocytes involved = 3 [22]. In total 314 patients could be evaluated for both liver histology and *IL28B* genotype.

### Fibrosis index

APRI was calculated as the ratio of normalized aspartate aminotransferase, i.e. value divided by the upper limit of normal, to the platelet count as previously detailed [23].

### Statistical methods

Wilcoxon-Mann-Whitney U-test, Kruskal-Wallis test, and Chi squared (χ^2^) tests were utilized to evaluate relationships between groups. All statistical analyses were performed using IBM SPSS statistics version 19.0 (IBM Corporation, Somers, NY) and StatView (version 5.0, SAS Institute Inc., Cary, NC, USA) software packages. All reported p-values are two-sided, and p-values<0.05 were considered significant.

### Ethical considerations

Written informed consent was obtained from each participating patient. Ethics committees in each participating country approved the study (i.e. Regional Ethical Review Board, Gothenburg, Sweden (Regionala etikprövningsnämnden i Göteborg), Regional Committee for Ethics in Medical Research, Bergen, Norway (Regionaletisk komite for medisinsk og helsefaglig forskning i Bergen), The Scientific Ethical Committee for the Region of Middle Jylland, Viborg, Denmark (Den Videnskabsetiske Komité for Region Midtjylland), The Scientific Ethical Committee for the Region of South Denmark, Vejle, Denmark (Den Videnskabsetiske Komité for Region Syddanmark), and the Ethics Committee, Department of Medicine for the Hospital District of Helsinki and Uusimaa, Finland (Etiska kommittén för invärtesmedicin)). The study has been registered at the NIH trial registry (ClinicalTrials.gov Identifier: NCT00143000).

## Results

**Figure 1:**
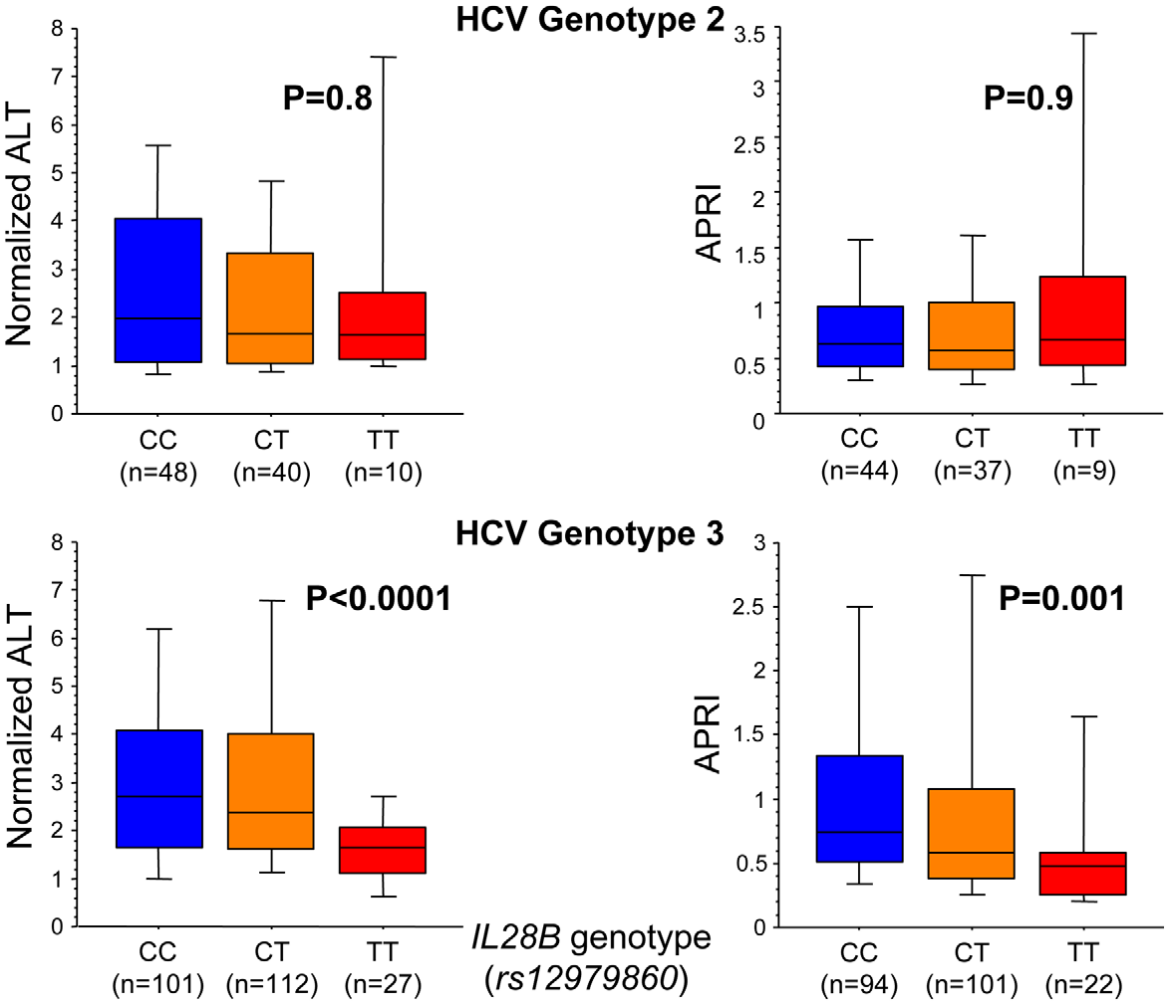
Impact of *IL28B* (*rs12979860*) on normalized ALT and AST to platelet ratio index (APRI) among HCV genotype 2 and 3 infected patients. Box plots displaying the 10^th^, 25^th^, 50^th^, 75^th^, and 90^th^ percentiles and p-values obtained using Kruskal-Wallis test.

**Figure 2:**
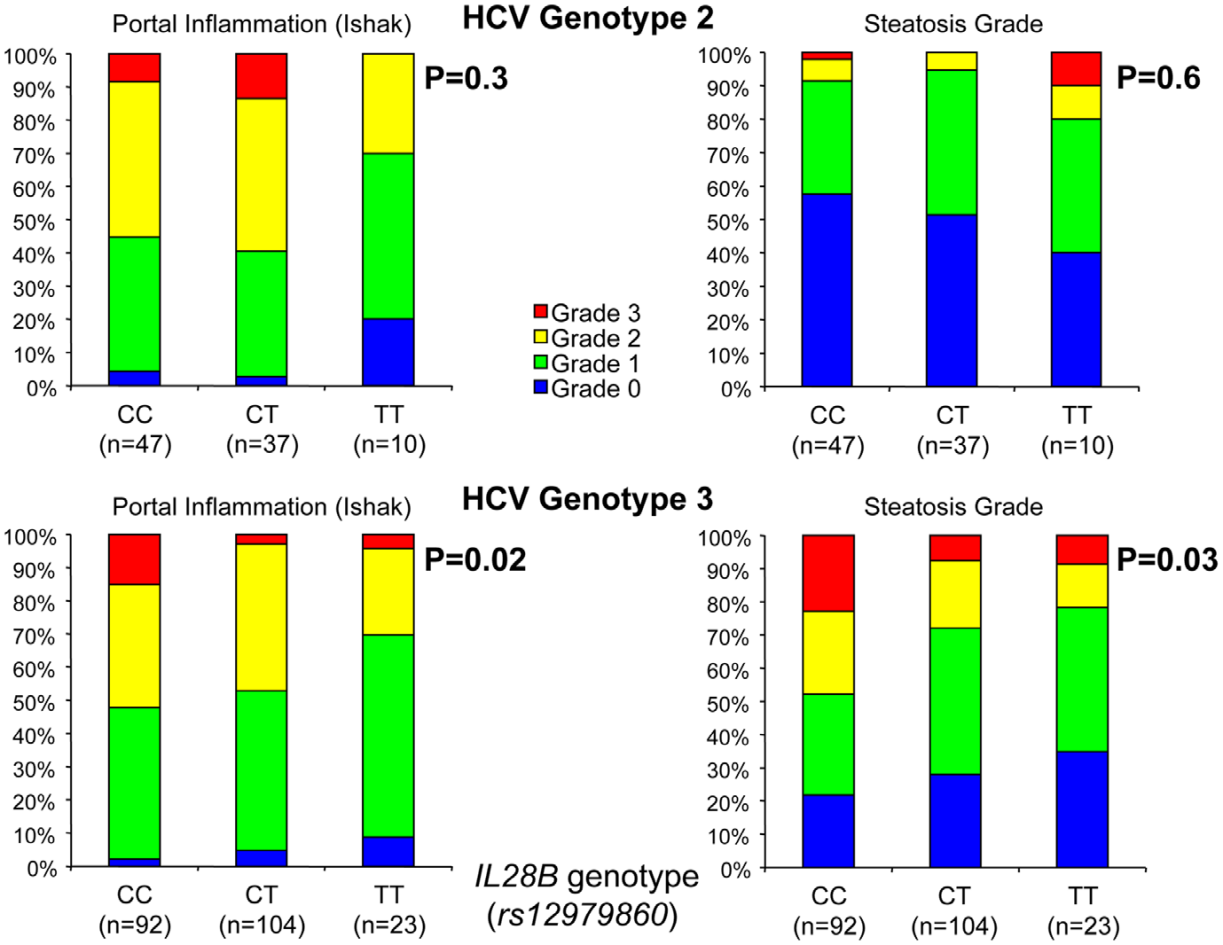
Association between *IL28B* (*rs12979860*) and portal inflammation grade (Ishak protocol) and steatosis grade among HCV genotype 2 and 3 infected patients. Histogram displaying the proportion of patients, and p-value obtained using Chi-squared test.

A strong association was noted between *IL28B* genotype and pretreatment normalized ALT (median 2.4, 2.3, and 1.6 for CC, CT, and TT respectively) and as well as APRI, a biomarker of liver fibrosis [23], (median 0.7, 0.6, and 0.5 for CC, CT, and TT respectively) among HCV genotype 3 infected patients, with homozygous TT carriers having the lowest levels (p<0.0001 and p = 0.001 respectively, Kruskal-Wallis test; Figure 1). With regards to ALT, CT carriers were more similar to CC than TT (CC vs. CT p = 0.6, CT vs. TT p = 0.0001, and CC vs. TT p<0.0001, Mann-Whitney U-test), but when evaluating APRI, CT patients were intermediary (CC vs. CT p = 0.03, CT vs. TT p = 0.07, and CC vs. TT p = 0.0015, Mann-Whitney U-test). This was mirrored by a significantly higher proportion of these patients having lower portal inflammation grades in their pretreatment liver biopsies, in contrast to homozygous CC carriers who had the highest grades (p = 0.02, Chi-squared test; Figure 2C). A similar non-significant trend was noted for interface hepatitis grade and fibrosis stage, with CC carriers having the highest grades and stages. No such trend was noted for the grade of lobular inflammation or confluent necrosis. In accordance with the observation for portal inflammation, homozygous CC carriers had the most pronounced steatosis and homozygous TT carriers had the least (p = 0.03, Chi squared test; Figure 2D). Because APRI is a continuous variable in contrast to fibrosis stage, which is ordered categorical data derived from liver biopsies prone to sampling error, different statistical methods are applicable, which may explain the significant association between *IL28B* genotype and APRI for genotype 3 in contrast to a non-significant trend for fibrosis stage.

The associations between *IL28B* genotype and ALT, APRI, portal inflammation grade, and steatosis observed among HCV genotype 3 infected patients, were not significant among HCV genotype 2 infected patients nor were any such trends noted among these latter patients. It should, however, be noted that the HCV genotype 2 infected patients enrolled in the present study were significantly older than those infected with HCV genotype 3 (mean age 47.2 vs. 39.8 years for HCV genotype 2 and 3 respectively, P<0.0001, Mann-Whitney U-test), and the number of patients enrolled with HCV genotype 2 was smaller than genotype 3 (98 vs. 241).

**Figure 3:**
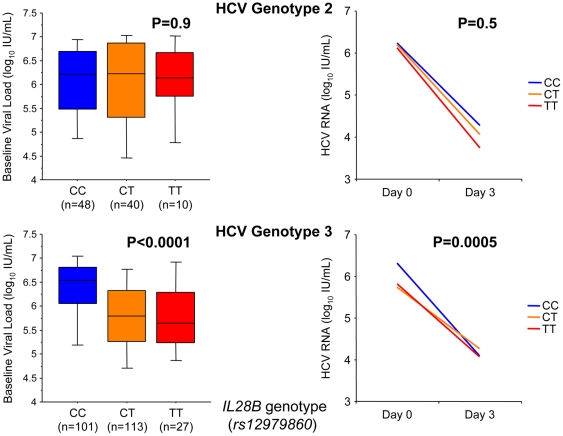
Impact of *IL28B* (*rs12979860*) on baseline viral load and decline in mean HCV RNA day 0–3 among HCV genotype 2 and 3 infected patients. Box plots displaying the 10^th^, 25^th^, 50^th^, 75^th^, and 90^th^ percentiles, and p-values obtained using Kruskal-Wallis test.

The HCV genotype 3 infected homozygous CC carriers had significantly higher baseline viral load (mean 6.3, 5.9, and 5.9 log10 IU/mL for CC, CT, and TT respectively, p<0.0001, Kruskal-Wallis test; Figure 3C) as well as first phase decline as measured by the decline in HCV RNA during the first three days of interferon/ribavirin therapy (mean 2.1, 1.7, and 1.9 log10 IU/mL for CC, CT, and TT respectively, p = 0.0005, Kruskal-Wallis test; Figure 3D). In contrast, no such associations were noted among HCV genotype 2 infected patients. Additionally no significant associations were noted among *IL28B* genotype and the likelihood of achieving SVR irrespective of HCV genotype. Further details regarding the impact of *IL28B* genotype on the viral kinetic during therapy in this trial are provided elsewhere [24].

**Figure 4:**
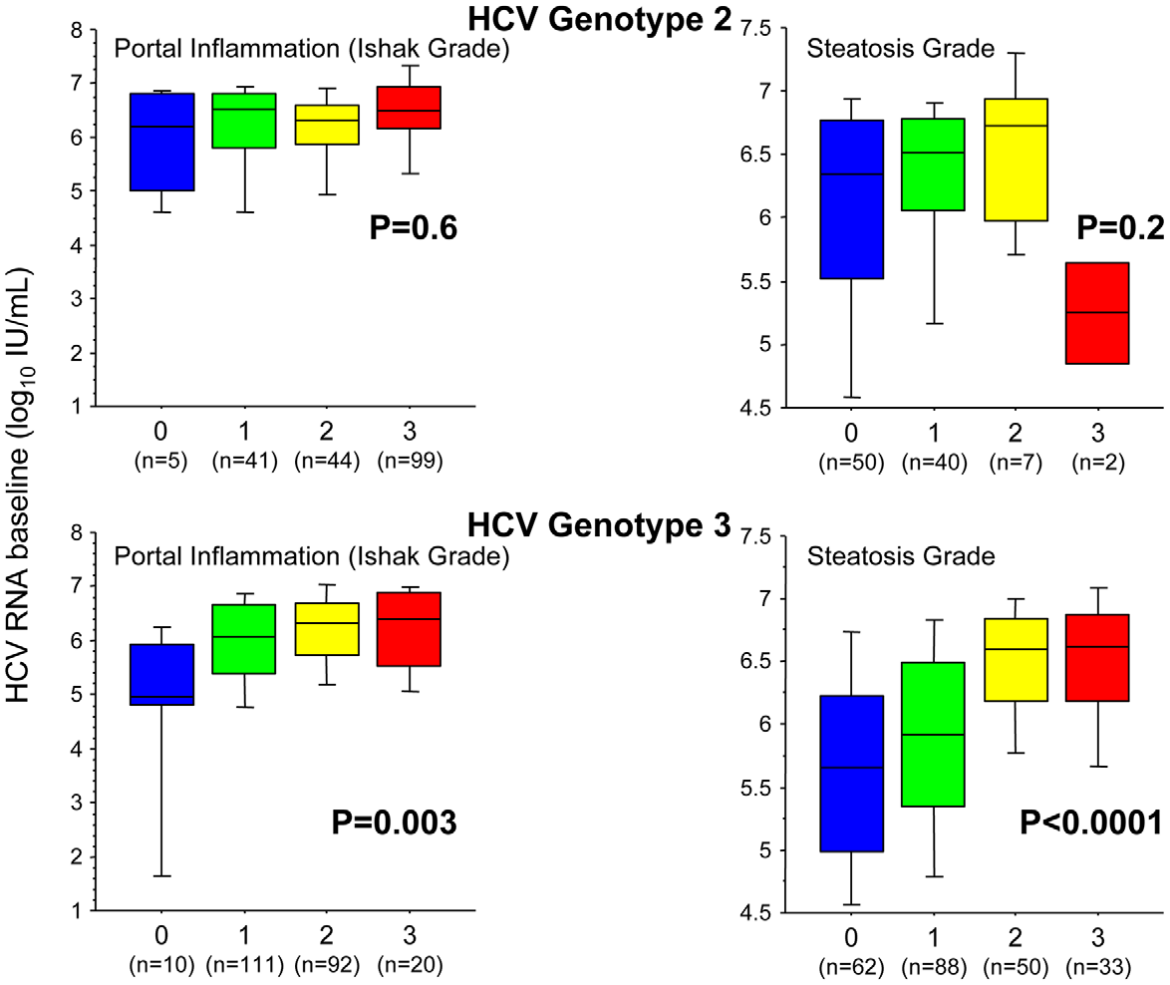
Association between baseline viral load and portal inflammation as well as steatosis among HCV genotype 2 and 3 infected patients. Box plots displaying the 10^th^, 25^th^, 50^th^, 75^th^, and 90^th^ percentiles, and p-values obtained using Kruskal-Wallis test.

Lower baseline HCV RNA levels were significantly associated with lower portal inflammation (p = 0.01, Chi-squared test) and steatosis grades (p<0.0001, Chi-squared test). Subgroup analyses revealed that these associations were significant for genotype 3 (p = 0.003 for portal inflammation and P<0.0001 for steatosis, Chi-squared test; Figure 4C and D respectively), but not for HCV genotype 2. These associations remained significant when controlling for IL28B genotype.

## Discussion

In spite of the beneficial treatment effects among patients carrying the C allele carriage at *rs12979860*, little is known regarding its impact on the natural history of chronic HCV. Abe *et al*. reported that among Japanese patients infected with HCV genotype 1 or 2, patients with homozygous carriage of the *IL28B* major allele had significantly higher inflammatory activity and higher fibrosis stage, indicating that this SNP genotype may not be beneficial outside the context of therapeutic intervention [18]. Similarly in a treatment trial by Moghaddam *et al.* involving HCV genotype 3 infected Caucasian patients, CC carriers at *rs12979860* had significantly higher normalized ALT levels and APRI scores than CT or TT carriers, however, liver biopsy evaluations were not reported [19]. In this context the main finding of the present study was a significant association between CC carriage at *rs12979860* and ALT, APRI score, portal inflammation grade, and steatosis among HCV genotype 3 infected patients, and that these associations appeared to be secondary to a significantly higher baseline viral load. Indeed it has previously been reported that among HCV genotype 3 infected patients, steatosis is more prevalent and pronounced than in other HCV genotypes [22], [25] due to impaired lipid export from infected hepatocytes [26], [27] possibly mediated by inhibition of microsomal triglyceride transfer protein (MTP) [28], [29] or due to increased availability of free fatty acids by reduced oxidation or by increased *de novo* synthesis [30], [31], [32], [33] of the genotype 3 core protein. Additionally the presence of steatosis in HCV genotype 3 infection is associated with higher baseline HCV RNA levels [25], [34], negatively impacts the clearance of HCV RNA during the initial 12 weeks of therapy [34], and entails accelerated fibrosis progression [22] indicative of a likely cytopathic effect of HCV genotype 3 virus. Moreover, it was recently reported that the cumulative mortality of HCV genotype 3 infected US Department of Veterans Affairs (VA) patients failing to achieve SVR after therapy was higher than among non-SVR patients infected with genotypes 1 or 2 [35] possibly secondary to HCV genotype 3 associated steatosis and ensuing liver damage.

In the setting of therapeutic intervention for HCV genotype 2 or 3, uncertainty prevails regarding the benefit of favorable *IL28B* allele carriage. Sarrazin *et al.* reported increased SVR rates following therapy among HCV genotype 2 or 3 infected Caucasian CC *rs12979860* carriers as compared to carriers of the T allele [36]. In contrast Mangia *et al.* noted an association between *IL28B* genotype and SVR only among HCV genotype 2 or 3 infected patients failing to achieve RVR [37]. Yu *et al.* reported a significantly higher rate of achieving RVR but not SVR among Asian homozygous TT *rs8099917* carriers infected with HCV genotype 2 [38] and Moghaddam *et al.* noted similar results among HCV genotype 3 infected Caucasian CC *rs12979860* carriers [19]. In the present study a significantly improved first phase decline in HCV RNA was noted among homozygous CC carriers infected with HCV genotype 3 but not among genotype 2. However, the higher baseline viral load among these genotype 3 infected patients essentially negated most of the favorable decline in HCV RNA observed during the initial three days of therapy. The reason for failing to observing an association between CC*_rs12979860_* and the first phase decline in HCV RNA among the HCV genotype 2 infected patients enrolled in the present study is not clear, but may indicate that *IL28B* SNP polymorphisms have a more pronounced therapeutic impact among HCV genotype 3 than genotype 2 infected Caucasian patients of Scandinavian descent.

In conclusion, the present study demonstrated an association between CC carriage at *rs12979860* and ALT, APRI, portal inflammation grade, and steatosis among HCV genotype 3 infected patients, and that this association may be secondary to the significantly higher baseline viral load. In this light, analysis of *IL28B* genotype may be beneficial among HCV genotype 3 infected patients so as to encourage homozygous CC *rs12979860* carriers to initiate therapy. Additionally the finding that *IL28B* variability did not impact on liver histopathology or viral load among genotype 2 infected patients implies that *IL28B* may differentially regulate the course of genotype 2 and 3 infection.

## References

[1] WHO (1999). Hepatitis C- globalprevalence (update).. Weekly Epidemilogical Report.

[2] Saito I, Miyamura T, Ohbayashi A, Harada H, Katayama T (1990). Hepatitis C virus infection is associated with the development of hepatocellular carcinoma.. Proc Natl Acad Sci U S A.

[3] Fried MW, Shiffman ML, Reddy KR, Smith C, Marinos G (2002). Peginterferon alfa-2a plus ribavirin for chronic hepatitis C virus infection.. N Engl J Med.

[4] Hadziyannis SJ, Sette H, Morgan TR, Balan V, Diago M (2004). Peginterferon-alpha2a and ribavirin combination therapy in chronic hepatitis C: a randomized study of treatment duration and ribavirin dose.. Ann Intern Med.

[5] Manns MP, McHutchison JG, Gordon SC, Rustgi VK, Shiffman M (2001). Peginterferon alfa-2b plus ribavirin compared with interferon alfa-2b plus ribavirin for initial treatment of chronic hepatitis C: a randomised trial.. Lancet.

[6] Rauch A, Kutalik Z, Descombes P, Cai T, Di Iulio J (2010). Genetic variation in IL28B is associated with chronic hepatitis C and treatment failure: a genome-wide association study.. Gastroenterology.

[7] Thomas DL, Thio CL, Martin MP, Qi Y, Ge D (2009). Genetic variation in IL28B and spontaneous clearance of hepatitis C virus.. Nature.

[8] Ge D, Fellay J, Thompson AJ, Simon JS, Shianna KV (2009). Genetic variation in IL28B predicts hepatitis C treatment-induced viral clearance.. Nature.

[9] Suppiah V, Moldovan M, Ahlenstiel G, Berg T, Weltman M (2009). IL28B is associated with response to chronic hepatitis C interferon-alpha and ribavirin therapy.. Nat Genet.

[10] Tanaka Y, Nishida N, Sugiyama M, Kurosaki M, Matsuura K (2009). Genome-wide association of IL28B with response to pegylated interferon-alpha and ribavirin therapy for chronic hepatitis C.. Nat Genet.

[11] Bochud PY, Bibert S, Negro F, Haagmans B, Soulier A (2011). IL28B polymorphisms predict reduction of HCV RNA from the first day of therapy in chronic hepatitis C.. J Hepatol.

[12] Darling JM, Aerssens J, Fanning G, McHutchison JG, Goldstein DB (2011). Quantitation of pretreatment serum interferon-gamma-inducible protein-10 improves the predictive value of an IL28B gene polymorphism for hepatitis C treatment response.. Hepatology.

[13] Lagging M, Askarieh G, Negro F, Bibert S, Soderholm J (2011). Response Prediction in Chronic Hepatitis C by Assessment of IP-10 and IL28B-Related Single Nucleotide Polymorphisms.. PLoS One.

[14] Honda M, Sakai A, Yamashita T, Nakamoto Y, Mizukoshi E (2010). Hepatic ISG expression is associated with genetic variation in interleukin 28B and the outcome of IFN therapy for chronic hepatitis C.. Gastroenterology.

[15] Dill MT, Duong FH, Vogt JE, Bibert S, Bochud PY (2011). Interferon-induced gene expression is a stronger predictor of treatment response than IL28B genotype in patients with hepatitis C.. Gastroenterology.

[16] McCarthy JJ, Li JH, Thompson A, Suchindran S, Lao XQ (2010). Replicated association between an IL28B gene variant and a sustained response to pegylated interferon and ribavirin.. Gastroenterology.

[17] Montes-Cano MA, Garcia-Lozano JR, Abad-Molina C, Romero-Gomez M, Barroso N (2010). Interleukin-28B genetic variants and hepatitis virus infection by different viral genotypes.. Hepatology.

[18] Abe H, Ochi H, Maekawa T, Hayes CN, Tsuge M (2010). Common variation of IL28 affects gamma-GTP levels and inflammation of the liver in chronically infected hepatitis C virus patients.. J Hepatol.

[19] Moghaddam A, Melum E, Reinton N, Ring-Larsen H, Verbaan H (2011). IL28B genetic variation and treatment response in patients with hepatitis C virus genotype 3 infection.. Hepatology.

[20] Lagging M, Langeland N, Pedersen C, Farkkila M, Buhl MR (2008). Randomized comparison of 12 or 24 weeks of peginterferon alpha-2a and ribavirin in chronic hepatitis C virus genotype 2/3 infection.. Hepatology.

[21] Ishak K, Baptista A, Bianchi L, Callea F, De Groote J (1995). Histological grading and staging of chronic hepatitis.. J Hepatol.

[22] Westin J, Nordlinder H, Lagging M, Norkrans G, Wejstal R (2002). Steatosis accelerates fibrosis development over time in hepatitis C virus genotype 3 infected patients.. J Hepatol.

[23] Wai CT, Greenson JK, Fontana RJ, Kalbfleisch JD, Marrero JA (2003). A simple noninvasive index can predict both significant fibrosis and cirrhosis in patients with chronic hepatitis C.. Hepatology.

[24] Lindh M, Lagging M, Farkkila M, Langeland N, Morch K (2011). Interleukin 28B Gene Variation at rs12979860 Determines Early Viral Kinetics During Treatment in Patients Carrying Genotypes 2 or 3 of Hepatitis C Virus.. J Infect Dis.

[25] Adinolfi LE, Gambardella M, Andreana A, Tripodi MF, Utili R (2001). Steatosis accelerates the progression of liver damage of chronic hepatitis C patients and correlates with specific HCV genotype and visceral obesity.. Hepatology.

[26] Rubbia-Brandt L, Quadri R, Abid K, Giostra E, Male PJ (2000). Hepatocyte steatosis is a cytopathic effect of hepatitis C virus genotype 3.. J Hepatol.

[27] Hofer H, Bankl HC, Wrba F, Steindl-Munda P, Peck-Radosavljevic M (2002). Hepatocellular fat accumulation and low serum cholesterol in patients infected with HCV-3a.. Am J Gastroenterol.

[28] Abid K, Pazienza V, de Gottardi A, Rubbia-Brandt L, Conne B (2005). An in vitro model of hepatitis C virus genotype 3a-associated triglycerides accumulation.. J Hepatol.

[29] Mirandola S, Realdon S, Iqbal J, Gerotto M, Dal Pero F (2006). Liver microsomal triglyceride transfer protein is involved in hepatitis C liver steatosis.. Gastroenterology.

[30] Jackel-Cram C, Babiuk LA, Liu Q (2007). Up-regulation of fatty acid synthase promoter by hepatitis C virus core protein: genotype-3a core has a stronger effect than genotype-1b core.. J Hepatol.

[31] Kawaguchi T, Yoshida T, Harada M, Hisamoto T, Nagao Y (2004). Hepatitis C virus down-regulates insulin receptor substrates 1 and 2 through up-regulation of suppressor of cytokine signaling 3.. Am J Pathol.

[32] Pazienza V, Clement S, Pugnale P, Conzelman S, Foti M (2007). The hepatitis C virus core protein of genotypes 3a and 1b downregulates insulin receptor substrate 1 through genotype-specific mechanisms.. Hepatology.

[33] Waris G, Felmlee DJ, Negro F, Siddiqui A (2007). Hepatitis C virus induces proteolytic cleavage of sterol regulatory element binding proteins and stimulates their phosphorylation via oxidative stress.. J Virol.

[34] Westin J, Lagging M, Dhillon AP, Norkrans G, Romero AI (2007). Impact of hepatic steatosis on viral kinetics and treatment outcome during antiviral treatment of chronic HCV infection.. J Viral Hepat.

[35] Backus LI, Boothroyd DB, Phillips BR, Belperio P, Halloran J (2011). A Sustained Virologic Response Reduces Risk of All-Cause Mortality in Patients With Hepatitis C.. Clin Gastroenterol Hepatol.

[36] Sarrazin C, Susser S, Doehring A, Lange CM, Muller T (2011). Importance of IL28B gene polymorphisms in hepatitis C virus genotype 2 and 3 infected patients.. J Hepatol.

[37] Mangia A, Thompson AJ, Santoro R, Piazzolla V, Tillmann HL (2010). An IL28B polymorphism determines treatment response of hepatitis C virus genotype 2 or 3 patients who do not achieve a rapid virologic response.. Gastroenterology.

[38] Yu ML, Huang CF, Huang JF, Chang NC, Yang JF (2011). Role of interleukin-28B polymorphisms in the treatment of hepatitis C virus genotype 2 infection in Asian patients.. Hepatology.

